# Environmental drivers of rabies in the Volga region of Russia: application of the maxent model

**DOI:** 10.3389/fvets.2025.1650834

**Published:** 2025-11-10

**Authors:** Olga I. Zakharova, Fedor I. Korennoy, Elena A. Liskova, Tatiana N. Demidova, Ivan V. Iashin, Andrei A. Blokhin

**Affiliations:** 1 Federal Research Center for Virology and Microbiology, Nizhny Novgorod, Russia; 2 Federal Centre for Animal Health (FGBI ARRIAH), Vladimir, Russia

**Keywords:** rabies, foxes, environmental factors, Maxent model, Russia, Volga region, zoonotic diseases

## Abstract

**Introduction:**

Understanding the dynamics of rabies virus spread in wild populations is essential for experts working to developing strategies to that protect ecosystems and prevent conflicts between wild and domestic animals. This is particularly important in the context of increasing human-wildlife interactions. Predictive modeling serves as a valuable tool for understanding and managing rabies in a given region. Such models not only aid in the prevention of outbreaks but also help optimize resource allocation for disease control and surveillance. Investigating abiotic factors that influence the incidence of rabies can further enhance the effectiveness of management strategies and reduce the associated risks to humans, livestock, and wildlife.

**Materials and methods:**

The aim of this study was to model rabies outbreaks and predict areas at high risk of new outbreaks among wild animals, based on climatic, landscape, and socio-demographic risk factors. To identify high-risk areas for rabies in wild animals using the ecological niche modeling approach, a dataset was compiled that included records of rabies outbreaks, as well as climatic and socio-demographic variables, including fox population density in the Volga region of the Russian Federation.

**Results:**

As a result, an ecological niche model for rabies outbreaks among wild animals was developed, incorporating the most significant variables for the region, with an accuracy of AUC = 0.85. Among the analyzed factors, climatic and landscape variables were found to be the most influential in determining the spread of rabies in wild populations. The most significant predictors included average annual temperature, population density, temperature seasonality, soil type, isothermality, and vegetation type. The model predicts that regions such as Nizhny Novgorod Oblast, the Republic of Mordovia, the Republic of Chuvashia, Penza Oblast, Saratov Oblast, and Samara Oblast are at high risk of rabies spread among wild animals.

**Conclusion:**

Thus, using ecological niche modeling, key risk factors for rabies were identified, and a geographical zoning of the Volga region was performed according to the level of risk of rabies transmission in wild animal populations. This spatial delineation has fundamentally transformed the approach to rabies management. Instead of applying uniform measures across the entire region, veterinary services can now implement a targeted strategy. This includes prioritizing intensifying wildlife surveillance in these areas, thereby optimizing the use of limited resources and enhancing the overall effectiveness of rabies control programs.

## 1 Introduction

Rabies is a viral zoonosis and is a multifaceted disease or impacts both animals and humans, presenting a significant threat to global public health and welfare (1–3). Rabies control programmes have been implemented in various countries to manage the disease in domestic animals, and many of these have achieved notable success. However, there remains a recognized need to address rabies transmission by wild animals, a topic that is often overlooked and receives insufficient attention (4–6).

The rabies virus belongs to the genus *Lyssavirus*, family *Rhabdoviridae* (4, 5). Transmission typically occurs through bites, scratches, or exposure to saliva with mucous membranes. The genus includes multiple species that are classified according to genetic and antigenic differences, with limited cross-protection among different phylogroups following vaccination (6, 7). Therefore, the study of this disease and the implementation of control measures, such as vaccination, are of critical importance for both public and veterinary health (7–10).

Rabies control programmes have been implemented in various countries to manage the disease in domestic animals, and many of these have achieved notable success. Rabies exists in two main forms: urban areas and sylvatic pattern. Sylvatic pattern of rabies is maintained in wildlife reservoirs such as foxes, wolves, skunks, mongooses, bats, and wild felids. Transmission to humans and domestic animals occurs through contact with these species, often resulting in “spillover” events (11–13).

Key strategies for rabies prevention and control in endemic areas include vaccination of both wild and domestic animal populations, as well as targeted disease control measures among wildlife. Vaccination campaigns for wild animals are typically carried out seasonally, with the aim of achieving at least 70% population coverage (14, 15). In many endemic regions, rabies control efforts appropriately focus on domestic animals—particularly dogs—since the majority of human rabies deaths are dog-mediated and interventions targeting dogs are considered the most cost-effective (12).

Despite this, the role of wild animals as rabies reservoirs, even in urban areas, is often underestimated. Researchers around the worldwide study are investigating multi-host transmission mechanisms, which are essential for understanding the epidemiology and developing effective control strategies. However, many aspects—such as cross-species transmission barriers and environmental drivers—remain poorly understood and require further systematic study (16, 17). A wide range of tools, from pattern analysis to next-generation sequencing (NGS) technologies, is used in these studies. Mathematical modeling is a powerful tool for analyzing the dynamics of infectious disease spread and for developing and optimizing surveillance and control strategies (18–20).

Statistical models based on regression equations, stochastic processes, and other methods allow for the analysis of complex interactions between pathogens, host populations, and the environment, making them essential for informed decision-making in public health and veterinary medicine. The analysis of changes in the spatial distribution and transmission dynamics of pathogens is a highly effective method for predicting epizootic situations (17). In the case of transmissible and zoonotic diseases, the transmission and spread of pathogens are closely linked to the ecological niche of the vector species or animal reservoirs (18, 21, 22). Therefore, it is believed that the origin and foci of rabies are correlated with the distribution of wild animals (23). As a result, the geographic distribution of infectious animal diseases can be predicted using ecological niche models (21).

Maximum entropy modeling (Maxent) is considered one of the most effective non-ensemble methods for ecological niche modeling. It is particularly well-suited for studies using occurrence-only presence data (22). Recently, this method has been successfully applied to predict the spread and transmission trends of emerging infectious diseases (24, 25).

In the study by Escobar et al. (25), it was demonstrated that niche modeling can be used to predict the distribution of infection foci among both wild and domestic animals, taking into account biotic interactions between the pathogen and its host, as well as the influence of climatic factors. The authors emphasize the importance of incorporating biotic factors to improve the accuracy of predictive models and highlight the necessity of a multidisciplinary approach.

According to a systematic review by Lawrence et al. (26), the ecological niche method has been successfully applied to predict the emergence and spread of diseases, including vector-borne and zoonotic infections, through the analysis of biotic and abiotic factors, as well as the study of changes in the distribution of vectors.

As a modeling and forecasting tool for disease occurrence, the ecological niche method is still evolving, addressing the limitations of traditional models by focusing on organism interactions and environmental changes. This makes it particularly valuable in the context of climate and landscape change.

In this study, we applied predictive modeling using the ecological niche method to rabies outbreaks in the Volga region of Russia. We identified significant natural, climatic, and socio-demographic factors influencing rabies in wild animals and ranked the regions according to their risk of rabies emergence in wildlife.

## 2 Materials and methods

### 2.1 Study area

The Volga Federal Region (VFR, or Privolzh’ye) is a macroregion that encompassing 14 federal subjects (first-level administrative divisions) located in the eastern part of the European territory of Russia. The administrative center of the region is the city of Nizhny Novgorod. The total area of the region is 1,036,975 square kilometers, which accounts for about 6.06% of Russia’s total territory. The population of the region is 28.6 million, with a density of 27.6 people per square kilometer. The urban population constitutes 73% of the total, and the region includes 191 cities, five of which have populations exceeding one million.

The Volga River serves as a natural boundary that conditionally divides the region into two parts: the right (western) bank, which is generally higher and more elevated, and the left (eastern) bank, which is lower and flatter. The main geographical feature of the Volga Federal Region is its location within the Russian Plain. The right bank of the Volga is more diverse in terms of topography, with the Volga Upland being a particularly notable feature. This upland reaches a maximum elevation of 384 meters and is deeply dissected by the valleys of numerous rivers. The left bank of the Volga consists of low-lying terrain with a general southern slope. In some areas, this lowland contains hills and elevations reaching up to 250–300 meters, with the highest point in the region reaching 482 meters.

The climate of the Volga Region is generally moderately continental, becoming more strongly continental toward the interior, it is characterized by distinct seasonality with cold, snowy winters and warm to hot summers. The eastern parts of the region tend to be slightly colder than the western parts, where the climate is more continental in nature.

The region is ecologically diverse, with a range of ecological zones. In the southern part, the landscape is dominated by steppe and semi-desert zones. The northern part features a forest-steppe zone, which covers nearly the entire right bank of the Volga. In the western part of the Volga Upland, there is a zone of broad-leaved and mixed forests, covering less than 25% of the region’s territory. Deciduous tree species are predominant in these forested areas. In contrast, coniferous species dominate in the European taiga zone, which is located further north (https://www.eea.europa.eu/en/datahub/datahubitem-view/11db8d14-f167-4cd5-9205-95638dfd9618).

### 2.2 Rabies data

Animal rabies data were obtained from two official sources. The first source consisted of reports from regional veterinary services, which documented rabies cases in various animal species, including wild, domestic, and farm animals. These data were collected based on the identification of bite marks, abnormal behavior, or the discovery of dead or captured animals exhibiting clinical signs of rabies, followed by laboratory confirmation of the rabies virus. The second source included statistical veterinary reports from the Federal Service for Veterinary and Phytosanitary Surveillance, detailing the imposition of quarantine and other restrictive measures in areas where rabies foci were officially confirmed. Laboratory confirmation of rabies in animals was conducted in accordance with the national standard “GOST 26075–13 Animals. Methods of laboratory diagnosis of rabies” (GOST 26075–2013, 2014), using the direct fluorescent antibody test (dFAT) (27).

A positive result was defined as the presence of yellow-green fluorescence in granules observed under a fluorescence microscope. In this study, a rabies outbreak was defined as the confirmation of at least one rabid animal, within a geographically defined area (e.g., the boundaries of an outbreak, hunting farm, herd, individual animal, farm, or village) (28).

**Figure 1:**
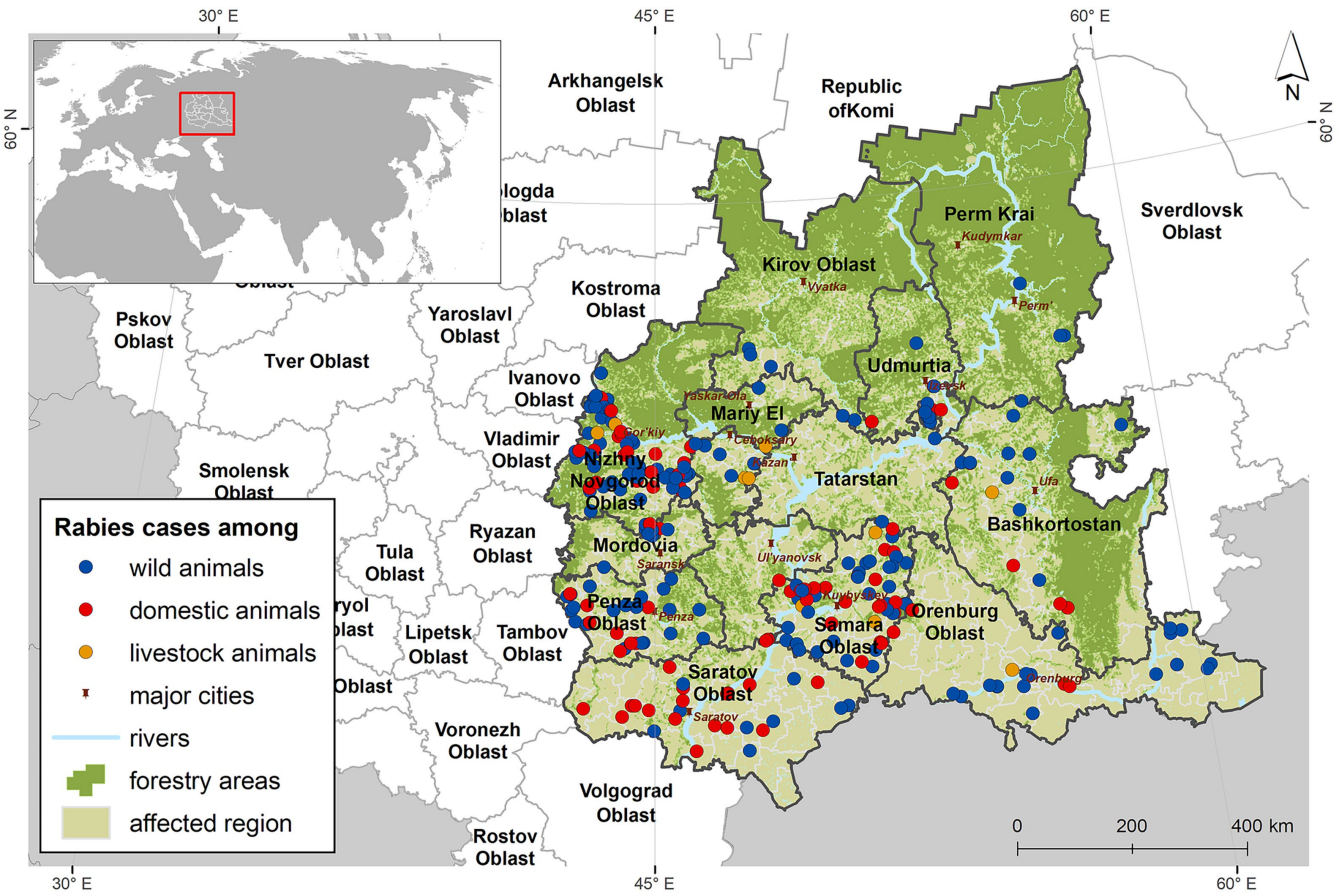
The Volga Region of Russia and animal rabies foci, 2012–2022 – 2024.

For each rabies focus, key attributes relevant to further modeling were extracted, including geographic coordinates, the species and number of infected animals, the start and end dates of quarantine measures. The geographic data were converted into shapefile format for visualization and further spatial modeling (Figure 1).

### 2.3 Climatic, environmental and landscape factors

A total of 27 variables of various types were used in simulations with the ecological niche model. Bioclimatic variables were obtained from the WorldClim database for the period 1970–2000, representing the “modern climate” (29). These variables are summarized in Table 1. Specifically, 11 bioclimatic variables were related to temperature (Bio01–Bio11), and eight were related to precipitation (Bio12–Bio19) (30). One variable represented altitude above sea level. In addition, two categorical variables represented vegetation and soil types (31).

**Table 1:** Variables, description and code used in the ecological niche model.

Variable/Code	Variable description	Data type
Alt	Altitude	Continuous
Bio01	Annual mean temperature −	Continuous
Bio02	Mean diurnal range [Mean of monthly (max temp − min temp)]	Continuous
Bio03	Isothermality (BIO2/BIO7) (×100)	Continuous
Bio04	Temperature seasonality (standard deviation ×100)	Continuous
Bio05	Max temperature of warmest month	Continuous
Bio06	Min temperature of coldest month	Continuous
Bio07	Temperature annual range (BIO5-BIO6)	Continuous
Bio08	Mean temperature of wettest quarter	Continuous
Bio09	Mean temperature of driest quarter	Continuous
Bio10	Mean temperature of warmest quarter	Continuous
Bio11	Mean temperature of coldest quarter	Continuous
Bio12	Annual precipitation	Continuous
Bio13	Precipitation of wettest month	Continuous
Bio14	Precipitation of driest month	Continuous
Bio15	Precipitation seasonality (Coefficient of variation)	Continuous
Bio16	Precipitation of wettest quarter	Continuous
Bio17	Precipitation of driest quarter	Continuous
Bio18	Precipitation of warmest quarter	Continuous
Bio19	Precipitation of coldest quarter	Continuous
Veg_bart	Vegetation map based on the use of daily S01 TOC Proba-V satellite data	Categorical
Wat_dist	Distance to main water bodies, m	Continuous
Soils	World soil information with a spatial resolution of 250 m	Categorical
Settl_dens	Density of settlements, units/km2	Continuous
Pop_dist	Distance to settlements, m	Continuous
Pop_dens	Population density, persons/km2	Continuous
Fox_dens	Fox population density, individuals/km2	Continuous

Socio-demographic variables included population density, settlement density, and fox population density.

Socio-economic data, including population density and settlement density, were obtained from the Federal State Statistics Service (Rosstat) (32).

Information on fox population distribution was retrieved from the Global Biodiversity Information Facility (GBIF) (https://www.gbif.org/). The dataset was preprocessed and formatted into an ASCII raster format for further analysis.

Soil-related variables were sourced from the Unified State Register of Soil Resources of Russia, which includes 255 soil units and is aligned with the globally harmonized soil database. The original vector data were converted into a raster format for compatibility with the modeling framework (33).

Distances to water bodies and settlements were calculated using a vegetation cover dataset and the Euclidean Distance tool in the ArcMap 10.8.2 geographic information system (ESRI, Redlands, CA, USA).

Land cover data were derived from a digital map based on satellite imagery from the Proba-V system, covering the period from 2000 to 2018. The original spatial resolution of the dataset was 100 × 100 meters (32).

Since the variables had different spatial resolutions, they were aggregated and resampled to a uniform spatial resolution of ~7 km^2^.

To avoid redundancy and ensure model reliability, an analysis of raster variables for multicollinearity was conducted. The analysis was performed using the **usdm** package in R, version 4.4.2 (33). Only variables with a Variance Inflation Factor (VIF) value of 10 or less were retained for further modeling. The workflow for multicollinearity testing included data preparation in ASCII (asc) format, followed by variable selection and the application of key functions from the usdm package: `vifcor`, `vifstep`, and `vif`. The process also involved setting threshold values, creating a reduced set of raster data, and performing validation. Upon inspection, we confirmed that all variables included in the VIF analysis had the same spatial resolution and extent. Thus, the usdm package enables the identification and removal of raster variables that exhibit strong multicollinearity based on VIF values. This process enhances the quality of subsequent ecological models and helps prevent biased or misleading results.

### 2.4 Modelling rabies suitability

The maximum entropy niche modeling (MaxEnt) method was applied to assess the relationship between rabies cases in wild animals and environmental variables. This approach, first described by Phillips and Dudík (22), is currently one of the most widely used methods for modeling the spatial distribution of a phenomenon based on presence-only data and risk factors. The underlying principle of the MaxEnt method is to find the probability distribution across sites that best matches the constraints imposed by the presence data—i.e., the distribution that maximizes information entropy (34). The resulting model provides a map showing the probability that the combination of environmental variables in each cell of the study area is suitable for the occurrence of the studied phenomenon.

In constructing the habitat suitability model for rabies outbreaks in the Volga region, we used 27 environmental variables, 19 of which described current climatic conditions. Two variables—soil type and land cover—were categorical. Presence data for the model inputs were represented as point occurrences of rabies outbreaks in wild animals. In total, the model evaluated 340 presence records, − of which 98.8% were in foxes, and 1.2% in raccoon dogs, wolves, and badgers. The number of pseudo-absences randomly selected for the model was 10,077. Model predictions were interpreted as habitat suitability indices (HSI), where 0 indicates completely unsuitable areas and 1 indicates completely suitable areas (35).

To assess the contribution of each variable to the prediction of habitat suitability and presence probability, we applied both the jackknife method and the heuristic variable contribution analysis provided by MaxEnt. The jackknife method evaluates the increase in AUC when a variable is used in isolation and the decrease in AUC when it is excluded from the full set of predictors. The heuristic method calculates the percentage contribution of each variable to the overall prediction of the distribution. These techniques enabled us to identify the most significant biological factors influencing rabies occurrence in the Volga region (36).

Model validation in MaxEnt was performed using dividing the presence data into 10 subsets (folds) without replacement. Each subset was used in turn for testing, and the final model results were based on the average values across the 10 replicates, along with standard deviation ranges. In each replication, 5,000 iterations were performed to achieve maximum gain with a convergence threshold of 0.00001.

To account for potential sampling bias associated with the overrepresentation of data near human settlements, we included a “bias” parameter expressed as populated places density. This reflects the likelihood that rabies cases are more frequently recorded in or near urban, town, and village areas. The density was calculated using the Kernel Density tool in GIS.

The predictive power of the model was assessed based on its ability to distinguish between presence and pseudo-absence data, and was quantified using the area under the receiver operating characteristic (ROC) curve (AUC). This metric reflects the probability that a randomly selected presence point has a higher predicted value than a randomly selected pseudo-absence point.

Using zonal statistics, the territory of each federal subject within the Volga region was classified into one of three risk zones based on the proportion of cells exhibiting maximum habitat suitability:

- Low risk (Zone 1): up to 10% of cells with high suitability
- Medium risk (Zone 2): 10–50% of cells with high suitability
- High risk (Zone 3): more than 50% of cells with high suitability

The resulting habitat suitability map was classified into low, medium, and high-risk zones using the quantile method, which divides the range of predicted values into three classes of equal frequency.

The risk of rabies occurrence at the level of administrative territories was ranked based on the proportion of spatial cells with a suitability score above 50% (i.e., “high-risk cells”) for each territory, taking into account environmental variables. These territories were subsequently categorized into three risk classes based on the proportion of high-risk cells (37, 38).

### 2.5 Software

Statistical data processing was performed in the MS Office Excel package (Microsoft, Redmond, WA, USA). Data preparation for analysis was performed the statistically oriented R software version 4.4.2. (39). Preprocessing of raster files and visualization of results was done in Geographic Information Systems ArcMap 10.8.2 and ArcGIS Pro 2.0.0. (ESRI, Redlands, CA, USA). Maximum entropy modeling was performed by means of MaxEnt software (35).

## 3 Results

### 3.1 Descriptive analysis

Between 2022 and 2024, a total of 345 rabies cases in animals were recorded in the Volga region. Of these, 340 cases were included in the epidemiological analysis, as five records were exluded due to missing data. The average annual number of confirmed rabies cases among wild animals was 47 (95% CI: 26–67), domestic animals 12 (95% CI: 8–37), and farm animals 6 (95% CI: 5–17).

**Figure 2:**
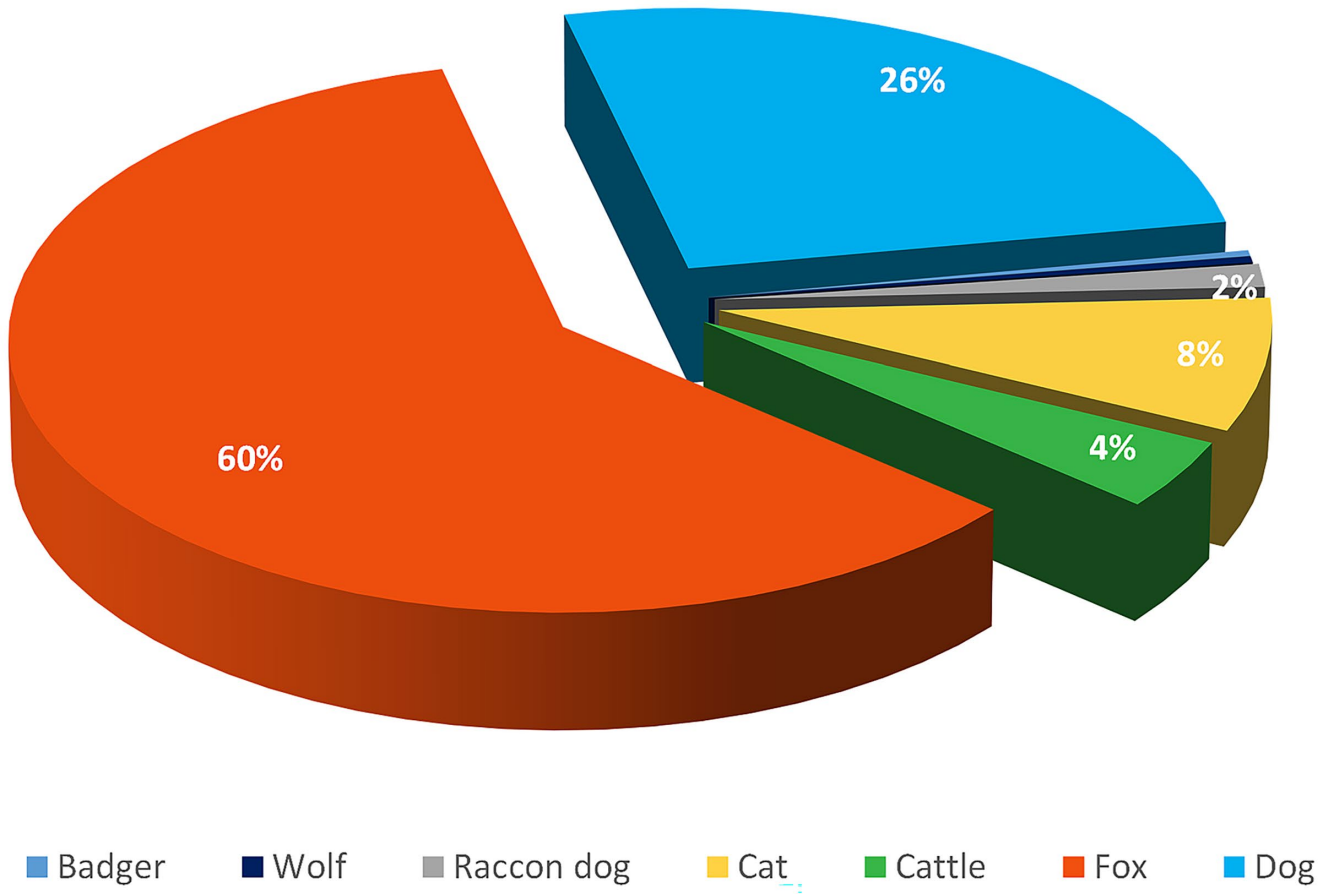
Distribution of rabies foci among different animal species in the Volga region of Russia from 2022 to 2024.

Among the recorded cases, 60% were in red foxes, 26% in dogs, and 8% in cats. Rabies foci in livestock, rabies cases were observed in cattle, accounting for 4% of the total number of cases. In wildlife, rabies was also reported in raccoon dogs, wolves, and badgers, each representing 0.4% of the total cases, but these occurrences were sporadic (Figure 2).

Analysis of rabies foci in the Volga region revealed, it became evident that the occurrence of the disease varied regionally, with local conditions influencing its distribution. A notable characteristic was the higher prevalence of rabies in areas with greater population density.

The average annual number of rabies outbreaks among wild animals varied across regions: 45 (95% CI: 8–65) in Nizhny Novgorod Oblast, 28 (95% CI: 6–34) in Samara Oblast, and 14 (95% CI: 3–20) in Saratov Oblast.

In regions of the Volga Federal District with lower population density, rabies foci in wild animals were predominantly sporadic, although a seasonal pattern was still evident.

In the Republics of Mordovia, Chuvashia, and Mari El, as well as in Kirov Oblast, rabies cases were reported sporadically throughout the 2022–2024 period.

In Penza Oblast, Orenburg Oblast, the Republic of Bashkortostan, and the Udmurt Republic, the average annual number of rabies foci among wild animals ranged from 8 to 25 (95% CI: 7–36).

**Figure 3:**
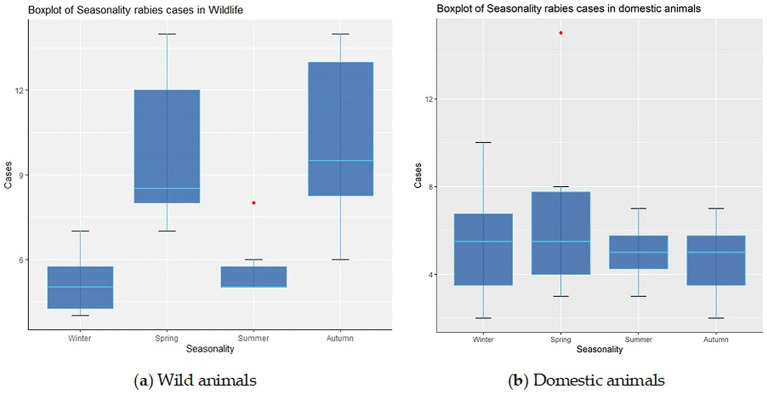
Distribution of rabies foci among wild animals **(A)** and domestic animals **(B)** by seasons of the year in the Volga region of Russia.

The seasonality of rabies outbreaks in both wild and domestic animals is illustrated in Figures 3a,b.

A tendency toward an increase in the registration of rabies outbreaks in wild animals increased in spring (median 9, IQR: 8–12) and autumn (median 10, IQR: 8–13).

During the winter and summer seasons, a decrease in the number of rabies cases was observed, with an average of 5 to 6 foci among wild animals, respectively (IQR: 4–6 for winter and IQR: 5–7 for summer).

Rabies cases in domestic animals also exhibited seasonal patterns, though without the sharp peaks or pronounced surges observed in wildlife. However, the analysis revealed a slight increase in the number of rabies foci in domestic in spring - 6 cases on average (IQR: 4–8) – compared with winter (5, IQR: 4–7), summer (4, IQR: 3–5) and autumn (5, IQR: 4–6) in the summer and autumn, respectively.

### 3.2 Modeled rabies suitability

The ecological niche model, implemented using the maximum entropy method via the MaxEnt software, demonstrated strong discriminatory power in distinguishing between true presence and pseudo-absence data, with an AUC value of 0.851 ± 0.018. This indicates a high level of model performance in predicting the spatial distribution of rabies foci.

**Figure 4:**
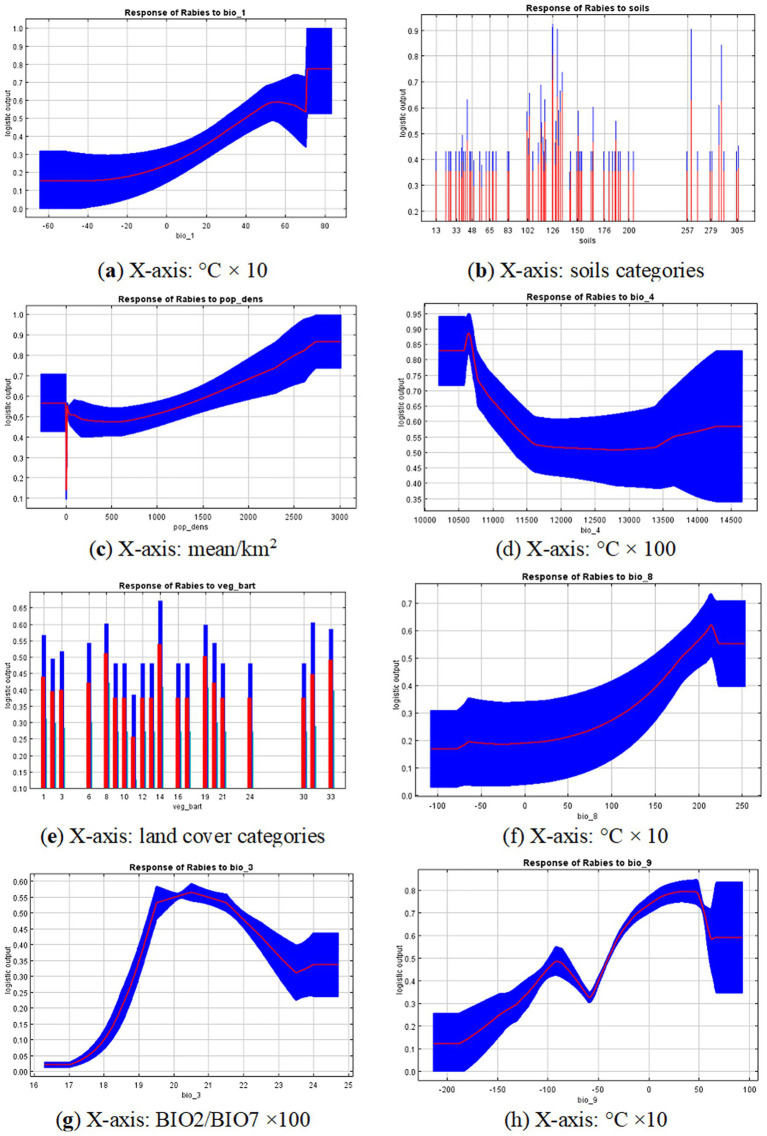
Response curves for the 8 variables that contribute the most: **(A)** Bio_1 - Mean annual temperature; **(B)** Population density; **(C)** Soils; **(D)** Bio_4 - Temperature seasonality; **(E)** Land cover type; **(F)** Bio_8 - Mean temperature of wettest quarter; **(G)** Bio_3 - isothermality; **(H)** Bio_9 - Mean temperature of driest quarter. Red lines represent average trends across 10 model replications and blue areas represent standard deviation limits. Y-axis is relative suitability calculated using the specific variable only. Units for x-axis variables are provided in the footnote of each graph.

The main variables that significantly contributed to the model fit for rabies foci in wild animals in the Volga region are presented in Figures 4a–h. The importance of these predictors was confirmed by the Jackknife test, which assessed the relative contribution of each variable to the model.

The most significant variables associated with the locations of rabies outbreaks in the Volga region included mean annual temperature, soil type, population density, temperature seasonality, vegetation type, mean temperature of the wettest quarter, and isothermality. The contribution percentages and permutation importance of these variables are presented in Table 2.

**Table 2:** Variable importance in ecological niche model of rabies in the Volga region of Russia.

Variable	Percent contribution	Permutation importance
Annual mean temperature, °C × 10	29.2	12.3
Soils of categorical	21.6	17.6
Population density, person/km^2^	13.1	12.9
Temperature seasonality (standard deviation ×100)	11.1	10.2
Vegetation of categorical	8.3	5.3
Mean temperature of wettest quarter	5.4	4.3
Isothermality (BIO2/BIO7) (×100)	2.7	10.5
Mean temperature of driest quarter	2.1	7.2
Altitude above sea level, m	2.0	7.3
Distance to populated areas, m	1.8	3.4
Precipitation of driest month	1.3	4.9
Precipitation seasonality (Coefficient of variation)	1.0	3.3
Distance to water bodies, m	0.6	0.9

The response curves of the variables indicate that certain areas within the study region are most suitable for the occurrence of rabies cases in wild animals. These areas are characterized by a mean annual temperature of 20 °C or higher, relatively high population densities (greater than 1,000 people/km^2^), and a temperature seasonality of about ±11.5 °C. The potential suitability of an area for rabies decreases as the deviation from these values increases. Additionally, suitable conditions were observed at a mean temperature of the wettest quarter of approximately 15 °C or higher, and at an isothermality of around 20%.

The land cover types most closely associated with the risk of rabies spread, as identified by the model, included urban and built-up areas, broadleaf forests, dark evergreen coniferous forests, open land and rocky outcrops, permanent wetlands, steppes, and cropland and pasture.

The soils in areas favorable for the habitation of red foxes—identified as the primary rabies virus carriers in the Volga region—were diverse. These included dark gray forest soils, podzolic soils with a second humus horizon, and deep gleyic soils. Additionally, the area included chestnut floury-carbonate soils without clear differentiation, as well as raised and degrading peat bogs. Solonetz (automorphic) soils, along with light chestnut solonetzic and solonchakous soils, were also present.

The remaining variables contribution less to the model but still affected the probability of rabies foci (Figure 4).

**Figure 5:**
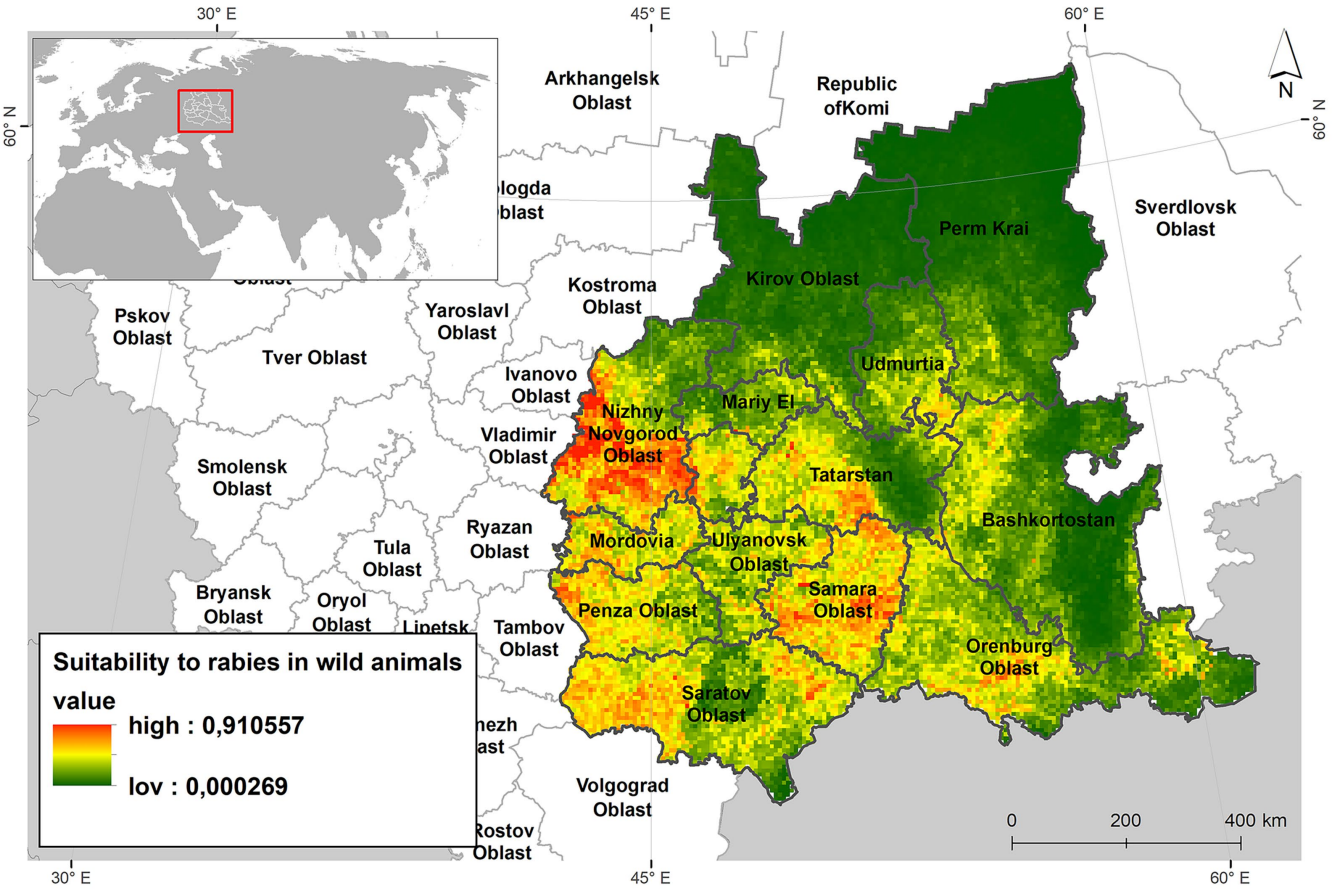
Suitability map for the red foxes niche habitats of ecological and demographic risk factors and current climate.

Figure 5 presents a habitat suitability map for the red fox, incorporating environmental, demographic risk factors, and current climatic conditions. The map clearly illustrates that the most suitable habitats are concentrated in the southwestern and southern parts of the Volga region—areas that also correspond to the most densely populated regions within the study area.

### 3.3 Risk map for rabies cases in wild animals

**Figure 6:**
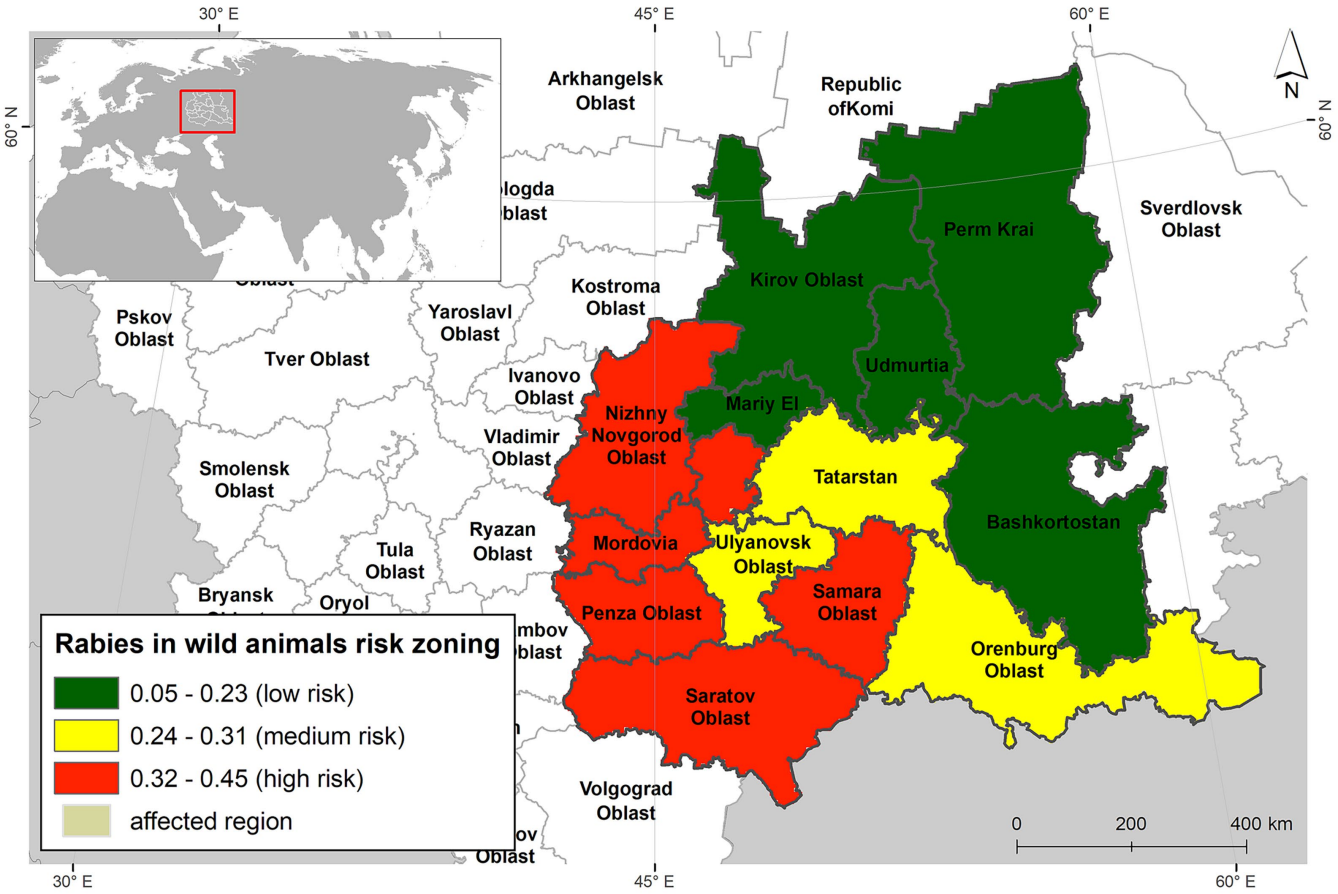
Risk map of rabies cases in wild animals of Volga region of Russia.

A conditional risk-level ranking was conducted based on the percentage of cells exhibiting the highest suitability (>50%) for the observed event—rabies foci in wild animals (Figure 6).

The low-risk zone included the northern federal subjects of the modeled region, such as Kirov Oblast, Perm Krai, and the Republics of Mari El, Udmurtia, and Bashkortostan. A medium risk of rabies foci was identified in the territories of Ulyanovsk Oblast, Orenburg Oblast, and the Republic of Tatarstan. The high-risk zone encompassed six federal subjects: Nizhny Novgorod Oblast, Penza Oblast, Samara Oblast, Saratov Oblast, and the Republics of Mordovia and Chuvashia.

## 4 Discussion

Rabies is a particularly dangerous disease because it serious threat to both animal and human health (6, 7).

Understanding the risk factors associated with rabies in animals is essential for comprehending the ecology and spatial dynamics of this disease. Comprehensive data on rabies outbreaks, along with detailed analyses and modeling, are crucial for developing effective strategies for disease control and prevention in wildlife. The elimination of rabies in wildlife is a complex and time-consuming process that requires an integrated approach, including vaccination programs, population monitoring, and public awareness campaigns. Therefore, a thorough understanding of rabies is the foundation for effective control of this disease (9, 40, 41).

The epidemiology of rabies in wildlife encompasses several key aspects that are essential for understanding the spread of the infection. It is important to identify factors that contribute to the transmission of the disease, such as host population density, migration patterns, and interspecies interactions (40, 42).

A descriptive analysis of rabies cases among animals in the Volga region revealed that the red fox is the primary species involved in the transmission of the rabies virus,

in 60.0 ± 5.0% of all cases. Domestic animals, particularly dogs and cats, followed in second and third place, with 26 ± 4.0% and 8 ± 2.0% of cases, respectively. Other wild animals accounted for less than 1% of all rabies cases.

The study of the seasonality of rabies foci is also a critical component, as the incidence of the disease varies according to environmental conditions that influence the behavior and activity of animals throughout the year. As a zoonotic disease, rabies in wild animals in the Volga region display clear seasonal patterns. For example, the highest number of rabies cases in foxes across all federal subjects of the Volga region was recorded during the spring and autumn seasons.

The seasonality of rabies incidence is closely related to the biological cycles of wild animals, including the breeding and dispersal of young. These processes increase the likelihood of encounters between wild and domestic animals, thereby facilitating the spread of the virus (43, 44).

Many wild species, such as foxes and raccoon dogs, have specific breeding seasons, which typically occur in the spring. During this period, animal activity is at its peak. In late spring and summer, following the birth and feeding of young, the dispersal of newly formed groups of wild animals occurs. Young individuals may move to new territories in search of food and shelter, increasing the likelihood of contact with infected animals (45).

In our study of rabies in the Volga region, we aimed to determine the relationship between rabies incidence and environmental variables and to construct predictive maps illustrating the suitability of the territory for the emergence of rabies among wild animals. To achieve this, we employed the ecological niche modeling approach using the maximum entropy method (MaxEnt) (46).

As a result, we identified several environmental and socio-economic factors associated with the geographic distribution of rabies. Environmental factors by high importance to development of natural focal diseases, as they directly affect pathogens or influence the number and distribution of hosts and vectors, thereby creating favorable conditions for the persistence and transmission of diseases (47, 48).

Among the most correlated environmental factors with of rabies outbreaks among wild animals in the Volga region establishing were mean annual temperature, mean temperature of the wettest quarter, temperature seasonality, and soil types. Mean annual temperature is a related shaping the ecosystem conditions in which wild foxes live. Changes in these conditions can significantly affect fox populations and behavior. Red foxes (*Vulpes vulpes*) are adaptable to a wide range of climates, but their distribution is limited by extreme temperatures. In our study, a mean annual temperature of 20 °C was identified as optimal for rabies occurrence in wild animals, particularly red foxes. A decrease in this temperature range may reduce the availability of suitable habitats. For example, warming trends may allow foxes to expand their range northward into previously inaccessible areas due to cold climates. Mean annual temperature also influences the availability of food for foxes. Warmer climates may alter the populations of rodents and other prey species that foxes rely on. Warm winters may increase pest populations, while cold winters may reduce food availability. Temperature changes can also affect nesting and shelter selection, with foxes potentially favoring warmer, sheltered locations during periods of suboptimal temperatures (49, 50).

While soils do not directly cause rabies, their characteristics can indirectly affect the likelihood of rabies occurrence and transmission through ecosystem interactions. Soils influence vegetation and food availability for wild animals. More productive soils support greater plant diversity and, consequently, larger animal populations. This, in turn, can affect predator populations. For example, an increase in rodent populations—potential carriers of the rabies virus—can raise the risk of rabies transmission among predators (51–53).

Urbanization, which involves the development of new areas and the transformation of natural habitats, as well as agricultural practices that alter soil properties, can influence animal behavior and migration patterns. When animals move into new regions with different, this can create new opportunities for rabies virus transmission (54).

From a socio-economic perspective, our study incorporated factors such as population density, settlement density, and distance to settlements. The model identified population density as a statistically significant factor associated with reported rabies foci (55).

High population density correlated to an increase in the number of stray companion animals, particularly in urban areas with inadequate animal control systems. Stray dogs and cats can act as vectors for rabies. In densely populated areas, rabies vaccination campaigns are more challenging to implement due to the logistical difficulties of reaching and vaccinating all animals, requiring strict monitoring of both owned and stray animals (56).

Areas with high population density may experience an increase in rabies cases due to the proximity of human settlements to wildlife, which increases the likelihood of contact between wild and domestic animals. In urban environments, high population density may encourage wildlife such as foxes or raccoon dogs to move into residential areas in search of food and shelter. This can lead to an increase in rabies incidence among domestic animals and, in turn, among humans (57, 58).

The modeling of rabies foci in wildlife using the maximum entropy method enabled us to identify the main risk factors associated with the occurrence of rabies in the Volga region of Russia. Based on these findings, we developed a risk map that classifies territories into high, medium, and low-risk zones for predicted rabies outbreaks.

Six federal subjects—Nizhny Novgorod Oblast, Penza Oblast, Samara Oblast, Saratov Oblast, and the Republics of Mordovia and Chuvashia—were identified as high-risk areas (59). The medium-risk zone included the territories of Ulyanovsk Oblast, Orenburg Oblast, and the Republic of Tatarstan.

While the MaxEnt model provides valuable probabilistic outputs for identifying areas at potential high risk of rabies outbreaks in the Volga region, it is crucial to acknowledge the inherent uncertainty in these predictions. The model’s performance is contingent on the quality and spatial completeness of the available data on reported cases and environmental variables. In areas with sparse surveillance data, the model may extrapolate beyond the conditions represented in the training data, leading to predictions with lower reliability. Furthermore, regions classified as “high risk” based on probability values might, in fact, have a wide confidence interval around that estimate, indicating a lower degree of certainty. To strengthen the interpretation of our risk maps, we incorporated measures of uncertainty, such as the standard deviation of model replicates. This analysis reveals that while the overall spatial pattern of risk is robust, predictive certainty is higher in central parts of the Volga region with more comprehensive data. In contrast, some peripheral areas identified as high-risk exhibit greater uncertainty, highlighting them as priorities for targeted surveillance to validate model projections and improve future forecasts. Therefore, the presented risk maps should be interpreted as hypotheses guiding proactive measures rather than definitive boundaries, with the understanding that uncertainty is an integral part of the spatial forecasting process for infectious diseases like rabies.

Ecological niche modeling identified key rabies risk factors, allowing for a geographical zoning of the Volga region. It facilitates a targeted approach where veterinary services can intensify wildlife surveillance in specific risk zones, leading to more efficient resource allocation and a more effective rabies control program.

## 5 Conclusion

We applied the maximum entropy method to the Volga region, a macroregion of Russia, to investigate the role of environmental determinants in the spread of rabies in wild animals. This modeling approach enabled us to assess the significance of various environmental variables and identify them as potential risk factors for rabies in wildlife.

The method of conditional zoning by risk level has been recommended to the veterinary services of the Volga region as a foundation for targeted surveillance of rabies outbreaks in wildlife. It also provides a framework for effective management through preventive measures directed at the primary animal hosts of the virus in the environment.

The findings of this study can contribute to the improvement of disease surveillance and control in wildlife by identifying high-risk areas. This approach offers a valuable tool for further research on the ecology of rabies in Russia and supports evidence-based decision-making in rabies prevention and control strategies.

## Data Availability

The raw data supporting the conclusions of this article will be made available by the authors, without undue reservation.
